# The predictive value of PaO_2_/FIO_2_ and additional parameters for in-hospital mortality in patients with acute pulmonary embolism: an 8-year prospective observational single-center cohort study

**DOI:** 10.1186/s12890-019-1005-5

**Published:** 2019-12-10

**Authors:** Yan Wang, He Yang, Lisong Qiao, Zheng Tan, Jin Jin, Jingjing Yang, Li Zhang, Bao Min Fang, Xiaomao Xu

**Affiliations:** Department of Respiratory and Critical Care Medicine, Beijing Hospital, National Center of Gerontology, Beijing, 100730 People’s Republic of China

**Keywords:** Predictive value, PaO_2_/FiO_2_ ratio, Parameters, In-hospital mortality

## Abstract

**Background:**

Rapid stratification and appropriate treatment on admission are critical to saving lives of patients with acute pulmonary embolism (PE). None of the clinical prediction tools perform well when applied to all patients with acute PE. It may be important to integrate respiratory features into the 2014 European Society of Cardiology model. First, we aimed to assess the relationship between the arterial partial pressure of oxygen/fraction of inspired oxygen (PaO_2_/FIO_2_) ratio and in-hospital mortality, determine the optimal cutoff value of PaO_2_/FIO_2_, and determine if this value, which is quick and easy to obtain on admission, is a predictor of in-hospital mortality in this population. Second, we aimed to evaluate the potential additional determinants including laboratory parameters that may affect the in-hospital mortality.

We hypothesized that the PaO2/FiO2 ratio would be a clinical prediction tool for in-hospital mortality in patients with acute PE.

**Methods:**

A prospective single-center observational cohort study was conducted in Beijing Hospital from January 2010 to November 2017. Arterial blood gas analysis data captured on admission, clinical characteristics, risk factors, laboratory data, imaging findings, and in-hospital mortality were compared between survivors and non-survivors. The area under the receiver operating characteristic curve (AUC) for in-hospital mortality based on the PaO_2_/FiO_2_ value was determined, and the association between the parameters and in-hospital mortality was analyzed by using logistic regression analysis.

**Results:**

Body mass index, history of cancer, PaO_2_/FiO_2_ value, pulse rate, cardiac troponin I level, lactate dehydrogenase level, white blood cell count, D-dimer level, and risk stratification measurements differed between survivors and non-survivors. The optimal cutoff value of PaO_2_/FiO_2_ for predicting mortality was 265 (AUC = 0.765, *P* < 0.001). Only a PaO_2_/FiO_2_ ratio < 265 (95% confidence interval [CI] 1.823–21.483, *P* = 0.004), history of cancer (95% CI 1.161–15.927, *P* = 0.029), and risk stratification (95% CI 1.047–16.957, *P* = 0.043) continued to be associated with an increased risk of in-hospital mortality of acute PE.

**Conclusion:**

A simple determination of the PaO_2_/FiO_2_ ratio at <265 may provide important information on admission about patients’ in-hospital prognosis, and PaO_2_/FiO_2_ ratio < 265, history of cancer, and risk stratification are predictors of in-hospital mortality of acute PE.

## Background

Acute pulmonary embolism (PE) occurs frequently and may cause death or serious disability [1]. Most deaths in patients with shock occur within the first few hours after admission [2]; therefore, rapid stratification and appropriate treatment are critical to save patients’ lives. It has been a challenge to predict the outcome of patients with acute PE, and risk stratification of patients with acute PE may physicians identify patients who may benefit from additional surveillance or therapy [3, 4]. Unfortunately, none of the clinical prediction tools perform well when applied to all patients with acute PE. Of all the clinical prediction tools evaluated, the one proposed by the European Society of Cardiology (ESC) is the best at risk stratifying patients [5]. However, several studies have still shown that more than 50% of patients with acute PE are hemodynamically stable on admission but have a high risk of death according to clinical models [6–8]. Additionally, another research study that assessed the ability of the 2014 ESC model to predict 30-day death after acute PE showed that stratification of patients at intermediate risk requires further improvement [9]. As more studies support the hypothesis of PE severity as a clinical continuum, it is important to find more scores, parameters, or biomarkers that would enable more accurate risk stratification in patients with acute PE [10].

Hypoxemia is common in acute PE and a very important mechanism in the pathogenesis of PE that leads to an adverse outcome. Although arterial blood gas analysis has been extensively evaluated in the clinical diagnostic algorithm of acute PE, it is not a criterion for evaluating the disease risk stratification. Studies have shown that risk stratification of patients with PE can be improved by integrating respiratory features into the 2014 ESC model [11, 12]. Among the parameters used to evaluate hypoxemia, arterial partial pressure of oxygen (PaO_2_) was not suitable because most patients were supplied oxygen before blood gas analysis was performed. The PaO_2_/fraction of inspired oxygen (FIO_2_) ratio, which is measured by the arterial partial pressure of oxygen to fraction of inspired oxygen, has been used as criterion for acute respiratory distress syndrome (ARDS) categories [13]. It was postulated that this ratio could be used to predict outcome in ARDS and several diseases [14–18], but no study has assessed the predictive value of PaO_2_/FIO_2_ in patients with acute PE.

In this study, we planned to assess the associations of PaO_2_/FIO_2_ with the risk of in-hospital mortality using a large registry of patients with PE. First, we aimed to assess the relationship between the PaO_2_/FIO_2_ ratio and in-hospital mortality, determine the optimal cutoff value of PaO_2_/FIO_2_, and determine if this value, quick and easy to obtain on admission, is a predictor of in-hospital mortality in this population. Second, we aimed to evaluate the potential additional determinants including laboratory parameters that may affect in-hospital mortality.

## Methods

### Study design

This prospective single-center observational cohort study was conducted in Beijing Hospital from January 2010 to November 2017. Patients with symptomatic objectively confirmed PE were screened. The classification of patients with acute PE was based on the 2014 ESC guideline [4]. We included consecutive patients with acute PE, confirmed by lung scintigraphy or computed tomographic pulmonary angiography (CTPA); and patients with available data on the Simplified Pulmonary Embolism Severity Index (sPESI) score, right ventricular dysfunction (RVD), and serum troponin level, as well as data on in-hospital mortality. Exclusion criteria were patients who were currently enrolled in a therapeutic clinical trial with a blinded therapy or unable to be followed for 3 months, patients with acute myocardial infarction with an elevated troponin I (TNI) or creatine kinase (CK) level, and patients missing any of the variables necessary to calculate PaO_2_/FIO_2_.

This study was approved by the ethics committee of Beijing Hospital (approval notice number: 2013BJYYEC-024-01) and China-Japan Friendship Hospital. Informed consent was obtained from participants in accordance with the independent local ethics committee and institutional review board requirements.

### Data collection

Arterial blood gas analysis data were captured on admission. Various clinical parameters, including clinical characteristics, risk factors, laboratory data (troponin and brain natriuretic peptide levels), presence of concomitant deep vein thrombosis (DVT), blood count abnormalities, imaging findings, and in-hospital mortality, were compared between survivors and non-survivors. The primary outcome was in-hospital mortality. The presence of DVT was evaluated by venous ultrasonography of the leg veins in patients clinically suspected of having DVT.

PE risk stratification of the patients with complete data was classified according to the 2014 ESC risk stratification model. The sPESI score was assessed as previously described (1 point for each of the following: age > 80 years, systolic blood pressure < 100 mmHg, heart rate ≥ 110 beats·min-1, oxygen saturation < 90%, history of cancer, and congestive heart failure or pulmonary diseases) [6]. RVD was assessed by either echocardiography or computed tomography (CT) angiography. Based on echocardiographic findings, RVD was defined by the presence of at least one of the following: 1) right-to-left ventricle end-diastolic diameter ratio > 1 in the apical four-chamber view, 2) right-to-left ventricle end-diastolic diameter ratio > 0.6 in the parasternal long-axis or subcostal four-chamber view, and 3) right ventricle/right atrial pressure gradient >30 mmHg [19]. RVD was not considered to have acute onset in the presence of right ventricular wall thickness > 7 mm or documentation of right ventricle overload during previous examinations. At CT angiography, RVD was defined as a right-to-left maximum dimension ratio > 0.9 when measured in the two-dimension axial transverse images at the valvular plane [19].

### Statistical analysis

Data are expressed as a mean ± standard deviation or median with interquartile range (IQR), if the data were skewed for continuous variables, and percentages for categorical variables. The continuous variables were compared between the two groups using the Student t-test and Mann-Whitney U test if they were non-normally distributed. The normality of the continuous variables was checked using the Kolmogorov-Smirnov test. The categorical variables are expressed as a frequency and percentage, and they were compared using the chi-square test or Fisher exact test. A *P*-value <0.05 was considered statistically significant.

Receiver operating characteristic (ROC) curve analyses were performed to evaluate the predictive power between different PaO_2_/FIO_2_ ratio values and determine the differences of the areas under the ROC curves (AUCs). In order to determine optimal cutoff values of the best predictive PaO_2_/FIO_2_ ratio value, optimum threshold estimation was applied. All statistical analyses were conducted using SPSS software, version 22.0 (IBM Corp., Armonk, NY).

## Results

**Fig. 1:**
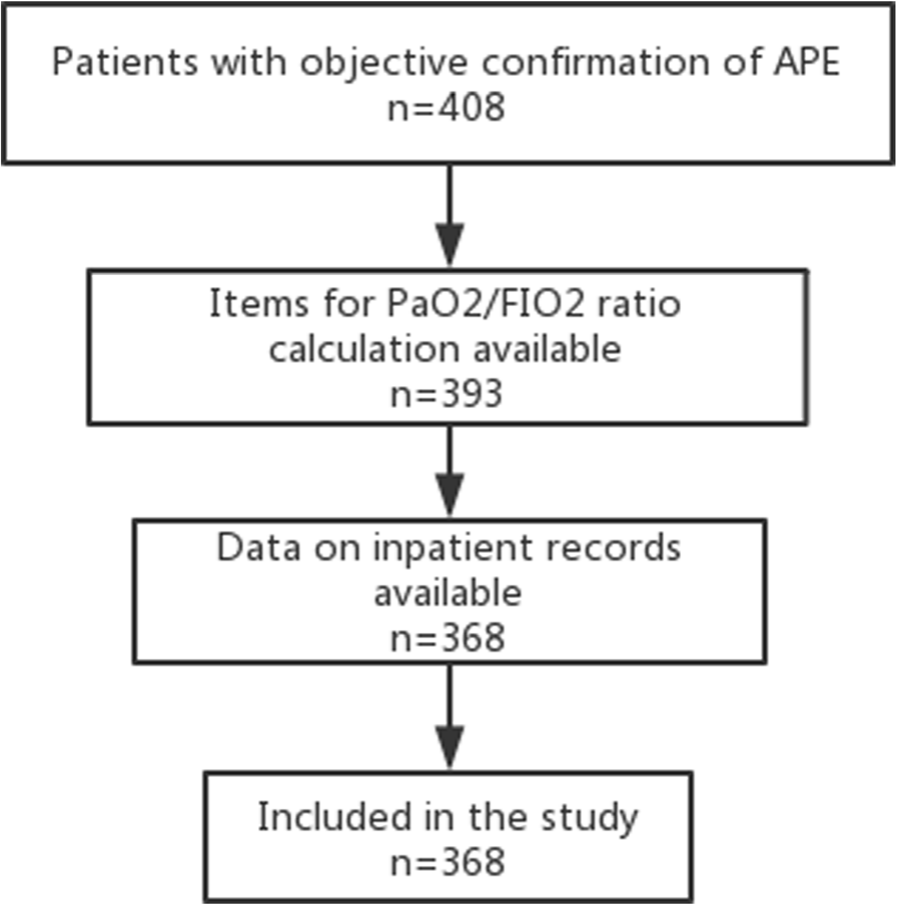
Flow diagram for inclusion of patients with acute pulmonary embolism (APE) in the final analysis

In this study, 408 patients were screened, 40 patients were excluded (25 had missing inpatient records, 2/25 were transferred to another hospital, and 15 lacked PaO_2_/FIO_2_ data), and 368 consecutive patients were included in the final analysis (Fig. 1). The median age of the cohort was 76 (IQR 65–83) years, and 179 patients (48.6%) were men. Complete baseline characteristics are presented in Table 1. Among the 368 patients diagnosed with acute PE by lung scintigraphy or CTPA during the study period, 317 (86.1%) had acute PE confirmed by contrast-enhanced CT, and 51 (13.9%) had acute PE confirmed by ventilation/perfusion lung scan. Three hundred twenty-nine patients (89.4%) underwent a sonographic examination of their leg veins. The groups were compared according to their demographic characteristic, medical history, and predisposing factor of PE. Those who survived to hospital discharge had a higher BMI (24.4 vs. 21.2 kg/m^2^, *P* < 0.001) and higher incidence of cancer (41 vs. 11, *P* < 0.001) than those who did not. There were no significant differences in medical history between the two groups.

**Table 1:** Baseline demographic and clinical characteristics of patients

	Survivors (*n* = 337, 91.6%)	Non-Survivors (*n* = 31, 8.4%)	Total (*n* = 368)	*P*-value
Male sex, n (%)	162 (48.1)	17 (54.8)	179 (48.6)	0.471
Age, years	75 (64–83)	79 (69–84)	76 (65–83)	0.096
BMI, kg/m^2^	24.4 (22–27)	21.2 (20.2–23.5)	24.2 (21.9–26.7)	0.000*
Medical history, n (%)				
Hypertension	172 (51.0)	17 (54.8)	189 (51.3)	0.686
Coronary heart disease	65 (19.2)	9 (29.0)	74 (20.1)	0.196
Heart failure	18 (5.3)	2 (6.4)	20 (5.4)	0.794
COPD	20 (5.9)	4 (12.9)	24 (6.5)	0.133
Diabetes mellitus	58 (17.2)	8 (25.8)	64 (17.3)	0.233
Chronic liver disease	2 (0.5)	1 (3.2)	3 (0.8)	0.119
Chronic renal disease	16 (4.7)	3 (9.6)	19 (5.2)	0.236
Predisposing factors, n (%)				
History of CVA	30 (8.9)	3 (9.6)	33 (9.0)	0.885
History of cancer	41 (12.1)	11 (35.5)	52 (14.1)	0.000*
Recent surgery	104 (30.8)	13 (41.9)	117 (31.8)	0.206
Immobilization >3 days	208 (61.7)	23 (74.2)	231 (62.8)	0.170
Varicosity	30 (8.9)	2 (6.5)	32 (8.7)	0.640
History of DVT	42 (12.4)	2 (6.5)	44 (12.0)	0.322
History of PE	51 (15.1)	3 (9.6)	54 (14.7)	0.409
History of trauma	22 (6.5)	1 (3.2)	23 (6.3)	0.466
Smokers	64 (19.0)	7 (22.5)	71 (19.3)	0.346

*BMI* Body mass index, *COPD* Chronic obstructive pulmonary disease, *CVA* Cerebral vascular accident, *DVT* Deep vein thrombosis, *PE* Pulmonary thromboembolism

### Clinical characteristic and laboratory findings

Table 2 shows the signs, symptoms, and laboratory findings of all patients, and there were no significant differences in symptoms between the two groups. Patients who survived to hospital discharge had a lower pulse rate than those who did not (80.1 ± 14.9 breaths/minute, *P* = 0.049). Compared with survivors, non-survivors were more likely to have a lower PaO_2_/FIO_2_ ratio (202 versus [vs.] 318.1, *P* < 0.001), higher D-dimer (1140 vs. 816.2 ng/mL, *P* = 0.002), higher LDH level (269 vs. 196 U/L, P < 0.001), higher cTNI level (0.05 vs. 0.01 ng/mL, *P* = 0.021), and higher white blood cell (WBC) count (9.3 vs. 6.5 × 10^9^/L, P < 0.001).

**Table 2:** Clinical characteristic of patients

	Survivors (*n* = 337, 91.6%)	Non-survivors (*n* = 31, 8.4%)	Total	*P*-value
Sign and symptoms, n (%)				
Dyspnea	205	20	225	0.687
Fever	49	3	52	0.458
Cough	128	13	141	0.665
Expectoration	105	13	118	0.219
Pleuritic chest pain	51	1	52	0.069
Syncope	27	2	29	0.758
Systolic blood pressure (mmHg)	129.2 ± 19.0	127.7 ± 17.6	129.0 ± 13.9	0.738
Pulse rate (beats/min)	80.1 ± 14.9	88.8 ± 19.6	81.0 ± 15.5	0.049*
Laboratory findings				
PaO_2_/FiO_2_ ratio on admission	318.1 (264.6–367.6)	202 (142.9–264.3)	311.4 (252.5–366.3)	0.000*
Hemoglobin level, g/L	124.7 ± 21.0	120.1 ± 27.7	125 (113–138)	0.269
White blood cell count, ×10^9^/L	6.5 (5.1–8.5)	9.3 (7.3–12.5)	6.82 (5.13–8.78)	0.000*
Platelet count, ×10^9^/L	207 (162–257)	198 (139–237)	206 (160–256)	0.142
D-dimer level, ng/mL	816.2 (285.5–1832)	1140 (846–2976)	856.0 (299.1–2004)	0.002*
CK level, U/L	55 (36–89)	42 (29–77)	54 (35–87)	0.286
LDH level, U/L	196 (164–243)	269 (216–455)	201 (165–253)	0.000*
cTNI level, ng/mL	0.01 (0.01–0.1)	0.05 (0.02–0.19)	0.02 (0.01–0.12)	0.021*
BNP level, pg/mL	185.7 (63.2–896.8)	211.6 (119.9–1297.2)	187.75 (66.03–936.6)	0.313

PaO2/FiO2 ratio, arterial partial pressure of oxygen/fraction of inspired oxygen ratio, *CK* Creatine kinase, *LDH* Lactate dehydrogenase; *cTNI* cardiac troponin I, *BNP* B-type natriuretic peptide

*p < 0.05

### Severity and outcome of patients

Table 3 shows the severity and outcome of the 368 patients, there are 337survivors and 31 non-survivors in this study, the in-hospital mortality is 8.4%(31/368). 345(314 survivors vs 31 non-survivors) patients provided data on the sPESI score, RVD, serum troponin and hemodynamic state for risk stratification according to the 2014 ESC guideline. There are 25 patients with unstable hemodynamic state belonged to the high risk group. Among haemodynamically stable patients (320 patients), sPESI 0 was found in 193 patients (60.3%) and sPESI ≥1 in 127 patients (39.7%). The poportion belonged to the high risk patients of the non-survivors is 35.5%(11/31). The in-hospital mortality for high-risk patients is 44%(11/25).There was a significant difference between survivors and non-survivors in risk stratification (*P* < 0.001) and length of hospital stay (17 vs. 10 days, *P* = 0.011).

**Table 3:** Severity and outcome of patients

	Survivors (*n* = 337, 91.6%)	Non-survivors (*n* = 31, 8.4%)	*P*-value
PE risk stratification	*n* = 314	*n* = 31	
High risk, n (%)	14 (4.5)	11 (35.5)	0.000*
Inter mediate risk, n (%)	118 (37.6)	9 (29.0)	
Low risk, n (%)	182 (58.0)	11 (35.5)	
Length of hospital stay, days, median (IQR)	17 (12–24)	10 (6–23)	0.011*

*PE* Pulmonary thromboembolism, *IQR* Interquartile range

*p < 0.05

### Optimal cutoff value of PaO2/FIO2 for predicting mortality

**Fig. 2:**
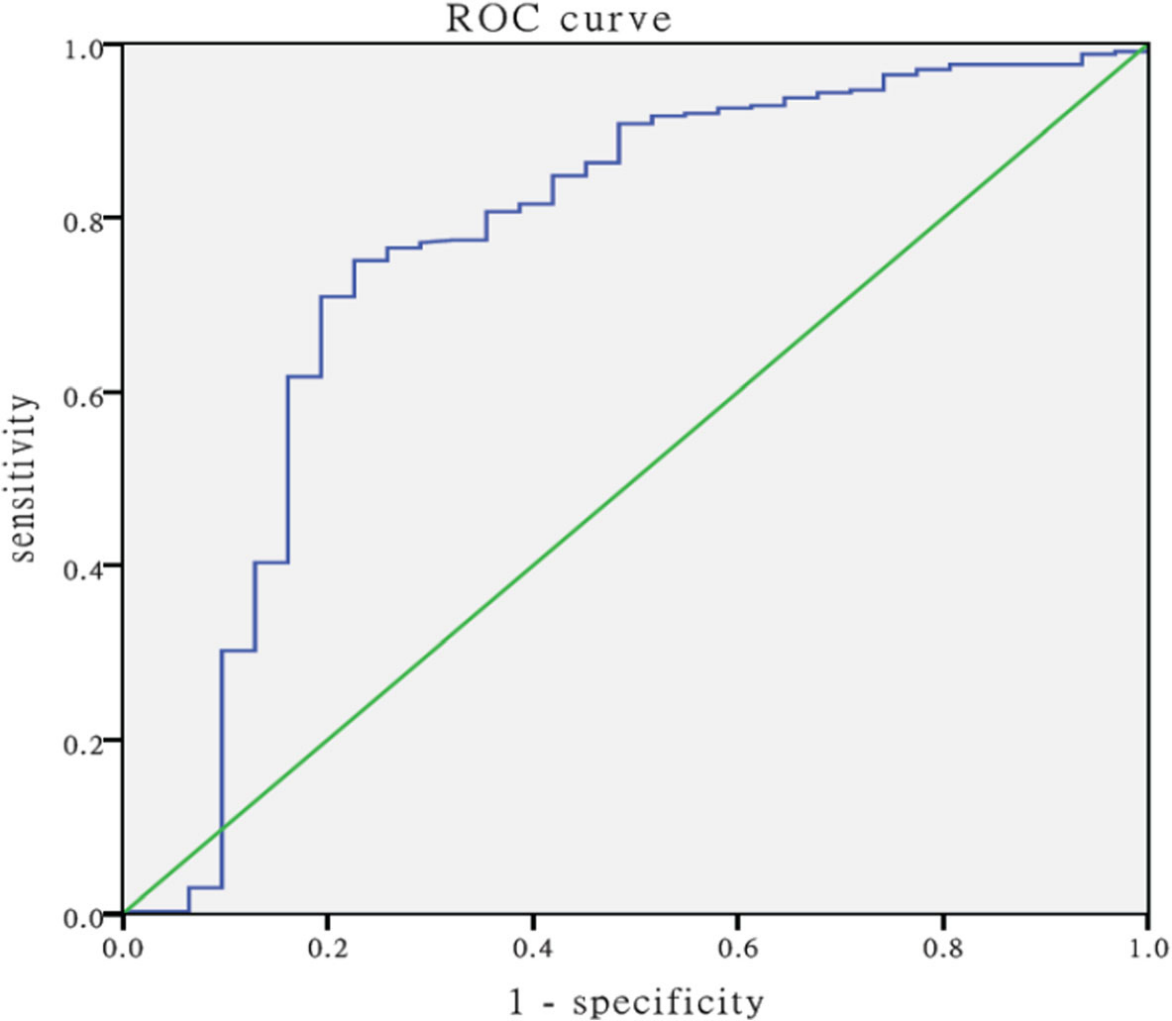
Receiver operating characteristic curve analysis of the PaO_2_/FIO_2_ on admission PaO_2_/FIO_2_, arterial partial pressure of oxygen and fraction of inspired oxygen

Figure 2 shows the optimal cutoff value of PaO_2_/FIO_2_ for predicting mortality was 265, with a sensitivity of 75.1%, specificity of 77.4%, and AUC of 0.765 (*P* < 0.001).

### Multivariable logistic regression for in-hospital mortality

Table 4 shows results from regression analyses. In the final multivariable model, after controlling for confounders, history of cancer (*P* = 0.029), PaO_2_/FIO_2_ ratio < 265 (*P* = 0.004), and risk stratification (*P* = 0.043) were associated with in-hospital mortality in patients with acute PE.

**Table 4:** Multivariable logistic regression analysis of in-hospital mortality

	OR	95% CI	*P*-value
BMI	0.945	0.824–1.084	0.421
Pulse rate	1.016	0.988–1.045	0.259
History of cancer	4.3	1.161–15.927	0.029*
PaO2/FiO2 < 265	6.310	1.823–21.483	0.004*
White blood cell count	1.098	0.947–1.275	0.216
D-dimer level	1.000	1.000–1.000	0.603
Elevated LDH level	1.992	0.670–5.921	0.215
cTNI level	0.847	0.56–1.28	0.430
Severity of PE stratification	4.214	1.047–16.957	0.043*

*OR* Odds ratio, *CI* Confidence interval, *BMI* Body mass index, *PaO2/FiO*_*2*_ Arterial partial pressure of oxygen/fraction of inspired oxygen, *LDH* Lactate dehydrogenase, *cTNI* cardiac troponin I, *PE* Pulmonary thromboembolism

*p < 0.05

## Discussion

In our study cohort, PaO_2_/FIO_2_ appeared to be associated with higher in-hospital mortality in patients with acute PE, and the optimal cutoff value of PaO_2_/FIO_2_ for predicting mortality was 265.

Although there was a significant difference between the survivors and non-survivors in only the pulse rate, there were no significant differences in other signs and symptoms, such as dyspnea, fever, cough, expectoration, etc. (Table 2). There was significant elevation of the WBC count in the non-survivors, which is in accordance with results of another study [20], which showed that the WBC count (OR, 1.9; 95% CI 1.2–3.5) predicted short-term (30-day) mortality following PE. There was a significant elevation of the D-dimer level in the non-survivors in this study Klok [21]. showed that high D-dimer levels were also correlated with centrally located pulmonary emboli and 15-day mortality, and there are also several studies evaluated the correlation between D-dimer levels and the burden of PE [22, 23]. We also found a significant difference between survivors and the non-survivors on the LDH level. Until now, there have been few studies about this biomarker. The cTNI was significantly increased in the non-survivors group in this study, Barrios [7] showed that elevated troponin levels were associated with a high all-cause mortality (OR, 4.3; 95% CI,2.1–8.5%), however, he also pointed out that troponin by itself did not appear to clinically significantly change the pretest to posttest probability of death, and the usefulness of basing therapeutic decision making solely on troponin levels does not appear warranted. So we combined all the above parameters with other prognostic instruments for PE.After adjusting for body mass index, history of cancer, PaO2/FiO2 ratio < 265, pulse rate, cTNI level, LDH level, WBC count, and D-dimer level, we found that the risk stratification, PaO2/FiO2 ratio < 265, and history of cancer continued to be associated with an increased risk of in-hospital mortality of acute PE (Table 4).

Acute PE impairs the efficient transfer of oxygen across the lung and leads to hypoxia, which is a very important mechanism in the pathogenesis of PE. Hypoxia is responsible for physiological consequences including but not limited to tachycardia, dyspnea, peripheral vasodilatation, and increased cardiac output. Hypoxia-mediated vasoconstriction is one of the causes of acute pulmonary hypertension, which is an important mechanism of acute right heart failure in PE [24]. These factors also lead to long-term outcomes like pulmonary hypertension or right ventricular failure [24]. However, hypoxia is not a criterion for evaluating disease risk stratification yet. Studies have shown that risk stratification of patients with PE can be improved by integrating respiratory features into the 2014 ESC model [11, 12]. Among the parameters used to describe hypoxia, PaO_2_ is the most common parameter, but it was not suitable for most patients with PE who were supplied oxygen on admission. One study on other parameters provided evidence that oxygen saturation or the respiratory rate could be added to the 2014 ESC strategy for risk stratification in order to further identify hemodynamically stable patients with PE at increased risk for death who are potentially candidates for more aggressive treatment [11]. Another study showed that both sPESI and pulse oximetry measurements are moderately accurate identifiers of low-risk patients with PE [25]. Although the PaO_2_/FIO_2_ ratio is widely used in the clinical setting for evaluating hypoxemia, there is some doubt about it [26, 27]. The PaO_2_/FiO_2_ ratio was used as a predictor of outcome in patients with ARDS, congenital diaphragmatic hernia, and cardiac surgery [14–18]. Studies also showed that the PaO_2_/FIO_2_ is not an independent predictor of mortality in patients with ARDS in multivariate analyses that controlled for other measures of severity of illness [28]. Because of the mechanism of right-to-left shunt, the variation of the PaO_2_/FIO_2_ ratio with an increasing FIO_2_ is complex [29]. It have been demonstrated that the PaO_2_/FIO_2_ ratio and its variation with changes in FIO_2_ depends on many clinical variables, and it may not be the only parameter for determining the state of arterial hypoxemia. In our cohort, PaO_2_/FIO_2_ ratio < 265 was related to the in-hospital mortality of patients with acute PE. Low values of the PaO_2_/FIO_2_ ratio may due to both the pathological conditions of the respiratory disease and alterations in the hemodynamic status of acute PE.

In our study, PaO_2_/FIO_2_ ratios were lower in patients at risk, so a simple determination of the PaO_2_/FIO_2_ ratio at <265 may provide important information about poor in-hospital prognosis. It is supposed that the hemodynamically stable patients with PaO_2_/FIO_2_ ratio < 265 may need more surveillance. According to the 2014 ESC model, high-risk PE is characterized by overt hemodynamic instability and the need for immediate advanced therapy, including consideration of fibrinolysis [30], and further study is warranted to determine whether the patients with a PaO_2_/FIO_2_ ratio at <265 are potentially candidates for more aggressive treatment.

### Limitations

The current study was a single-center observational study. However, our study is the largest prospective study to date that addresses the PaO_2_/FIO_2_ ratio for PE. Our results may be clinically relevant since a simple determination of the PaO_2_/FIO_2_ ratio may provide very important information for determining in-hospital mortality of patients with acute PE.

In addition, despite its single-center design, our results showed consistency with other respiratory features in previous reports related to the in-hospital mortality of acute PE. Hypoxia also leads to long-term effects of the compensatory mechanisms of PE, such as pulmonary hypertension or right ventricular failure; therefore, future studies need to determine whether these factors affect the long-term outcome of this corhort .

## Conclusions

In summary, the PaO_2_/FIO_2_ ratio may be useful for identifying in-hospital mortality of patients with acute PE on admission. PaO_2_/FIO_2_ ratios are lower in patients at risk, and the value of 265 is the cutoff point of predicting in-hospital mortality. A simple determination of the PaO_2_/FIO_2_ ratio at <265 may provide important information about patients’ in-hospital prognosis, and these patients may need more surveillance. Integrating respiratory features into the 2014 ESC risk stratification model of PE is also important, and future studies or clinical trials will be required to clarify the clinical utility of the PaO_2_/FIO_2_ ratio in a larger sample of patients.

## Data Availability

The datasets used and/or analysed during the current study are available from the corresponding author on reasonable request.

## Abbreviations

(sPESI) score: Simplified Pulmonary Embolism Severity Index
ARDS: Acute respiratory distress syndrome
AUC: Area under the receiver operating characteristic curve
CK: Creatine kinase
COPD: Chronic obstructive pulmonary disease
CT: Computed tomography
CTPA: Computed tomography pulmonary angiography
CVA: Cerebral vascular accident
DVT: Deep vein thrombosis
ESC: European Society of Cardiology
IQR: Interquartile range
PaO_2_/FIO_2_: Arterial partial pressure of oxygen/fraction of inspired oxygen
PE: Pulmonary embolism
ROC: Receiver operating characteristic
RVD: Right ventricular dysfunction
TNI: Troponin I
vs.: versus
WBC: white blood cell
